# Biosynthesis of Medium-Chain ω-Hydroxy Fatty Acids by AlkBGT of *Pseudomonas putida* GPo1 With Native FadL in Engineered *Escherichia coli*

**DOI:** 10.3389/fbioe.2019.00273

**Published:** 2019-10-17

**Authors:** Qiaofei He, George N. Bennett, Ka-Yiu San, Hui Wu

**Affiliations:** ^1^State Key Laboratory of Bioreactor Engineering, East China University of Science and Technology, Shanghai, China; ^2^Shanghai Collaborative Innovation Center for Biomanufacturing Technology, Shanghai, China; ^3^Key Laboratory of Bio-based Material Engineering of China National Light Industry Council, Shanghai, China; ^4^Department of Bioengineering, Rice University, Houston, TX, United States; ^5^Department of Chemical and Biomolecular Engineering, Rice University, Houston, TX, United States

**Keywords:** *Escherichia coli*, AlkBGT, medium-chain fatty acids, ω-hydroxy fatty acids, FadL

## Abstract

Hydroxy fatty acids (HFAs) are valuable compounds that are widely used in medical, cosmetic and food fields. Production of ω-HFAs *via* bioconversion by engineered *Escherichia coli* has received a lot of attention because this process is environmentally friendly. In this study, a whole-cell bio-catalysis strategy was established to synthesize medium-chain ω-HFAs based on the AlkBGT hydroxylation system from *Pseudomonas putida* GPo1. The effects of blocking the β-oxidation of fatty acids (FAs) and enhancing the transportation of FAs on ω-HFAs bio-production were also investigated. When *fadE* and *fadD* were deleted, the consumption of decanoic acid decreased, and the yield of ω-hydroxydecanoic acid was enhanced remarkably. Additionally, the co-expression of the FA transporter protein, FadL, played an important role in increasing the conversion rate of ω-hydroxydecanoic acid. As a result, the concentration and yield of ω-hydroxydecanoic acid in NH03(pBGT-*fadL*) increased to 309 mg/L and 0.86 mol/mol, respectively. This whole-cell bio-catalysis system was further applied to the biosynthesis of ω-hydroxyoctanoic acid and ω-hydroxydodecanoic acid using octanoic acid and dodecanoic acid as substrates, respectively. The concentrations of ω-hydroxyoctanoic acid and ω-hydroxydodecanoic acid reached 275.48 and 249.03 mg/L, with yields of 0.63 and 0.56 mol/mol, respectively. This study demonstrated that the overexpression of AlkBGT coupled with native FadL is an efficient strategy to synthesize medium-chain ω-HFAs from medium-chain FAs in *fadE* and *fadD* mutant *E. coli* strains.

## Introduction

Production of bio-based chemicals has received a lot of attention due to concerns around limited non-renewable fossil fuels and the global environment. Fatty acids (FAs) from plant oils are among the most abundant resources in nature (USDA, 2018). FAs are renewable industrial chemical feedstocks, and some artificial microbial pathways have been established to synthesize high-value chemicals based on FA utilization. These reactions in the microbial pathways are mainly related to double-bond generation, double-bond oxidation, double-bond cleavage, epoxidation and functionalization of C-H bonds in alkyl chains (Biermann et al., 2011). Hydroxy fatty acids (HFAs), one kind of FA derivative, are the direct products of FA hydroxylation via C-H bond oxygenation.

HFAs are valuable chemicals due to their hydroxyl functional group and carboxyl group. HFAs have also gained great interest from various industries because of their special properties: higher reactivity, solvent miscibility, stability, and viscosity. Therefore, HFAs have wide applications and can be used as pharmaceutical intermediates and synthetic precursors (Cross et al., 1995), cosmetic ingredients, surfactants, foam improvers, emulsified agents in deodorant sticks, etc. (Koay et al., 2011), and as food bio-preservatives because of their strong antifungal activity (Cheng et al., 2010). Additionally, ω-HFAs are the ideal building blocks of green synthetic fibers for polymer materials due to the location of hydroxyl groups on the terminus of long carbon chains, which provide superior material properties (Weng and Wu, 2008; Cao and Zhang, 2013). ω-Hydroxydecanoic acid can be further derivatized to sebacic acid, which is an important precursor in the production of nylon and polyamides (PAs), primarily 4,10-PA and 5,10-PA (Bowen et al., 2016). ω-Hydroxydodecanoic acid has the potential to enable commercially relevant production of C12 α, ω-DCA, a valuable precursor of nylon-6,12 (Sugiharto et al., 2018).

Currently, considerable effort has been expended to produce HFAs by chemical synthesis; however, there are still various problems, e.g., lack of environmentally friendly solutions, complicated extraction methods, poor selectivity and harsh reaction conditions (Liu et al., 2011). Bio-catalysis is therefore regarded as a promising alternative approach because it has several advantages such as high selectivity, broad substrate spectrum, high catalytic efficiency, mufti-step reaction in a single strain, and is environmentally friendly (Manfred et al., 2011; Lin and Tao, 2017). Among all ω-HFAs produced by C-H bond oxygenases, most ω-HFAs are usually produced *via* cytochrome P450 monooxygenase (CYP)-based platforms. For example, these include CYP52A13 and CYP52A17 (Craft et al., 2003; Eschenfeldt et al., 2003), CYP4 (Fer et al., 2008), CYP704B2 (Li et al., 2010), CYP102A1 (Xiao et al., 2018), CYP153 (Bordeaux et al., 2012), and the CYP153A_M.aq._-CPR_BM3_ fusion protein (Scheps et al., 2013; Ahsan et al., 2018; Joo et al., 2019). Some of these enzymes can ω-oxidize terminal methyl groups and have been applied to ω-HFA production, but there is a limitation, in that most of these enzymes are specific for chain lengths of ≥12 (Picataggio et al., 1992).

The alkane hydroxylase of *Pseudomonas putida* GPo1, which consists of three components (AlkB, AlkG, and AlkT), could be another notable ω-oxidation enzyme system of FAs. This system has high specificity for medium-chain fatty acid (C_5_-C_12_) oxidation at terminal positions (Grant et al., 2011; Manfred et al., 2011; Tsai et al., 2017). The mono-oxygenase encoded by *alkB* belongs to a large class of membrane-bound proteins and is an essential component of the alkane hydroxylase system (Grund et al., 1975; Menno et al., 1989; Beilen et al., 1992). Rubredoxin encoded by *alkG* and rubredoxin reductase encoded by *alkT* are two electron transfer proteins. The hydroxylase system *in vivo* operates as follows: AlkG transfers an electron from AlkT, which reduces its flavin adenine dinucleotide at the expense of NADH, then further delivers the electron to AlkB to achieve hydroxylation (Jan et al., 2002). As reported, the AlkBGT hydroxylation system has been investigated in the bioconversion of the fatty acids methyl ester and alkane (Grant et al., 2011; Manfred et al., 2011; Julsing et al., 2012; Call et al., 2016; van Nuland et al., 2016; Kadisch et al., 2017), but there are no reports on ω-HFA production.

*E. coli* is the ideal host to employ to exploit the hydroxylation activity of these enzymes because of its clarified metabolic background. FA metabolism in *E. coli* has been expounded in previous work. FA β-oxidation in *E. coli* is induced by the presence of FAs in the growth medium. The FA β-oxidation cycle is activated by acyl-CoA synthetase (FadD), which plays an important role in FA degradation. The catabolic oxidation cycle consists of dehydrogenation by FadE, hydration and dehydrogenation by FadB or FadJ, and thiolation with CoA by FadA or FadI (Lennen and Pfleger, 2012; Wu and San, 2014). Regulation of FA oxidation is performed by the control protein FadR, which represses the *fad* regulon. FadD and FadE are the critical enzymes associated with β-oxidation (Wang et al., 2015; Bule et al., 2016). An important approach is deletion of *fadD* and *fadE*, which block the breakdown of fatty acids into acyl-CoA (Steen et al., 2010; Handke et al., 2011). Cao et al. (2016) constructed a *fadD* knockout strain, and the engineered strain showed an enhanced ability to produce HFAs. The titer of HFAs reached 58.7 mg/L, which is 1.6-fold higher than that of the original strain. Kirtz et al. (2016) and Sung et al. (2015) observed improved production of ω-hydroxy fatty acids with a *fadD* knockout strain. In addition, FA transport through the cell membrane to reach intracellular enzymes is one of the hurdles in ω-HFA production. There are two approaches to overcome this hurdle: one is the addition of chemical reagents such as surfactants to enhance membrane permeabilization, and another is using a transporter protein. Many studies have been done with transporter proteins. For example, AlkL from *Pseudomonas putida* GPo1 was reported to transport alkanes and fatty acid methyl esters into *E. coli* but was not active for fatty acids (Jeon et al., 2018). In fact, the outer membrane FA transport mechanism was also clarified in *E. coli*. It was clearly demonstrated that FA transport is mediated by a membrane protein pump (Bonen et al., 2007). FadL is a typical transporter for FAs; it can use spontaneous conformational changes to transport hydrophobic substrates by diffusion (van den Berg, 2005). There have been many notable cases of the use of FadL to achieve increased production (Bae et al., 2014; Tan et al., 2017; Jeon et al., 2018; Wu et al., 2019).

In this study, the medium-chain alkane hydroxylation system, AlkBGT, from *P. oleovorans* GPo1 was introduced in *E. coli* strains for ω-hydroxydecanoic acid biosynthesis from decanoic acid (C10FA). Metabolic engineering strategies to reduce the fatty acid metabolism of the host strains were also investigated here. Blocking the β-oxidation of fatty acids by deletion of *fadE* and *fadD* highly increased the yield of ω-hydroxydecanoic acid. In addition, co-expression of the native FA transporter protein, FadL, enhanced the consumption rate of decanoic acid as well as the yield of ω-hydroxydecanoic acid. Octanoic acid and dodecanoic acid were also used as substrates in this hydroxylation system. The results showed that this established system has good efficiency for the synthesis of ω-hydroxyoctanoic acid and ω-hydroxydodecanoic acid. To our knowledge, the current study constitutes the first report of AlkBGT to be applied to FA hydroxylation, and it provides a generalizable framework for the production of medium-chain ω-HFAs via AlkBGT in engineered whole-cell systems. Meanwhile, this study provides a new platform for medium-chain ω-HFA production.

## Materials and Methods

### Strains and Plasmids

The strains and plasmids used in this study are listed in Table 1. *E. coli* DH5α was used for plasmid construction, and *E. coli* W3110 was used as the host and initial strain for further genetic manipulation. Ribosome binding sites (RBS) between *alkB-alkG* and *alkG-alkT* were calculated by online software (https://salislab.net/software/forward). The *alkB, alkG*, and *alkT* genes with optimized ribosome binding sites were cloned into pTrc99a, and the formed plasmid was named pBGT. The strains with *fadD* and *fadE* deletion were constructed using a CRISPR–Cas9 and λ Red recombination system-based genome editing system, which is composed of five elements: Cas9-expressing cassette, gRNA expression plasmid, λ Red recombination, donor template DNA, and inducible plasmid curing system for eliminating gRNA plasmid from the cells (Li et al., 2015). In brief, to construct the gRNA expression plasmid, the pGRB backbone was amplified by PCR with a pair of primers that included 20-bp spacer sequences specific for the target gene. The PCR product was then ligated via homologous recombination to obtain the desired gRNA expression plasmid. To construct donor dsDNA, a pair of 300–500-bp homologous sequences, which are upstream and downstream sequences of the target gene, were amplified by PCR separately and then fused together by overlapping PCR. The mutant strain was verified with genomic PCR after construction to ensure that the target gene had been deleted. For construction of the AlkBGT and FadL expression plasmids, the one-step cloning method was applied. The primers used in this study are listed in Table 2. Gene segments of *alkB, alkG, alkT* from *P. putida* GPo1 were amplified by PCR. Ribosome binding sites inside the *trc-alkB, alkB*-*alkG*, and *alkG*-*alkT* genes were calculated by online software version 2.0 (https://salislab.net/software/forward). The *alkB, alkG*, and *alkT* genes with optimized ribosome binding sites were cloned into pTrc99a, and the formed plasmid was named pBGT. The native *fadL* was further added behind *alkT* in pBGT with optimized ribosome binding sites, and the newly formed plasmid was named pBGT-*fadL*.

**Table 1:** Strains and plasmids.

**Strains and plasmids**	**Relevant characteristics**	**Source**
**Strains**		
*E. coli* DH5α	Wild-type	Laboratory collection
*E. coli* W3110	Wild-type	Laboratory collection
NH01	W3110 Δ*fadE*	This study
NH02	W3110 Δ*fadD*	This study
NH03	W3110 Δ*fadE* Δ*fadD*	This study
**Plasmids**		
pBGT	pTrc99a carries *alkB, alkG, alkT* from *Pseudomonas putida* P1	This study
pBGT-*fadL*	pBGT carries *fadL* from *E. coli*	This study
pGRB	Amp^R^, bla, gRNA expression vector	Li et al., 2015
pREDCas9	Spe^R^, Cas9, λ Red recombinase expression vector	Li et al., 2015

**Table 2:** Primers of plasmid and strain construction.

ptrc99aF	CCATGGAATTCGAGCTCGGTAC
H-ptrc99aR	CTCCTTACCCTTTTTGCGAATTGTTATCCGC TCACAATTCCACAC
AlkB-HF	CGCAAAAAGGGTAAGGAGGTATTATATGAATGG CAAAAGCAGCGTTC
AlkB-R	CTATGATGCTACCGCGGTTG
AlkG-HF	GCGGTAGCATCATAGCTACTATATAGAAAAGAGG AGGTAATTCATGGCTAGGTATCAGTGTCCAG
Alk-R	TTAGCCTAACTTTTCCTGATAGAGTACATAG
AlkT-HF	GAAAAGTTAGGCTAAACCTAATATAATTATTTTAAGGA GGAAAAACATGGCAATTGTTATTGTTGGCGCTG
AlkT-R	GAGCTCGAATTCCATCTAATCAGGTAATT TTATACTCCCGCCAAG
pBGT-F	TCTAGAGTCGACCTGCAGGC
pBGT-R	GGATCCCCGGGTACCGAGC
FadL-F	AGCTCGGTACCCGGGGATCCTTGACAATTA ATCATCCGGCTCGTAT
FadL-R	GCCTGCAGGTCGACTCTAGATCAGAACGCGTAG TTAAAGTTAGTACC
FadE-SF	GCCTGCAGGTCGACTCTAGACCGCCGACCCAATT CATCAG
FadE-SR	AGCTCGGTACCCGGGGATCCCTGATGAATTGGG TCGGCGG
FadE-D1F	AGGTGGAGATCCCCAGCAGTAC
FadE-D1R	CTTTCGGCTCCGTTATTCATAACGAAAAGCC CCTTACTTGTAGGAG
FadE-D2F	CAAGTAAGGGGCTTTTCGTTATGAATAACG GAGCCGAAAGG
FadE-D2R	CGTGTTATCGCCAGGCTTTAGG
FadD-SF	GCCTGCAGGTCGACTCTAGATCCAGTCT GCATCTTTCCGC
FadD-SR	AGCTCGGTACCCGGGGATCCGCGGAAAG ATGCAGACTGGA
FadD-D1F	AAGGGAAAACTCGCCTGGAA
FadD-D1R	TCTGACGACTGACTTAACGCTTCTTCAC CTCTAAAATGCGTGTTC
FadD-D2F	ATTTTAGAGGTGAAGAAGCGTTAAGT CAGTCGTCAGA
FadD-D2R	TAACAGATACCAGACATCCGC

### Media and Culture Conditions

The medium for strain construction and primary preculture was Luria-Bertani Broth (LB), containing tryptone 10 g/L, yeast extract 5 g/L, and sodium chloride 10 g/L. The modified M9 medium used for whole-cell bioconversion contained Na_2_HPO_4_·12H_2_O 15.12 g/L, KH_2_PO_4_ 3.0 g/L, NaCl 0.5 g/L, MgSO_4_·7H_2_O 0.5 g/L, CaCl_2_ 0.011 g/L, 1% (w/v) vitamin B1 0.2 ml/L, glucose 5 g/L, and trace elements solution 0.2 ml/L. The total amount of decanoic acid was ~500 mg/L. The trace elements solution contained (per liter): FeCl_3_·6H_2_O 5 g; MnCl_2_·4H_2_O 2 g; ZnCl_2_ 0.684 g; CoCl_2_·6H_2_O 0.476 g, CuCl_2_·2H_2_O 0.17 g; H_3_BO_3_ 0.062 g; Na_2_MoO_4_·2H_2_O 0.005 g.

To prepare the cell culture, the primary precultures were prepared by transferring one single colony to 3 ml of LB medium with a specific concentration of antibiotics (ampicillin 100 mg/L) at 37°C overnight. In the secondary precultures, 1 ml of the primary preculture was inoculated into 50 ml of LB medium at 37°C for 8 h. Then, a fresh 1 ml of the secondary preculture was transferred into 500-ml flasks containing 100 ml of LB medium with 2 g/L glucose at 30°C, 220 rpm. The expression of target genes was induced with IPTG when the OD_600_ reached 0.5–0.6, and the cells were grown for 12 h at 30°C. For whole-cell bioconversion, after 12 h of induction, the cells were harvested by centrifugation at 5,945 g and 4°C for 5 min. Then, the cells were washed twice with nitrogen-free M9 medium and resuspended in the modified M9 medium with 20 OD_600_, with the addition of 0.1% (vol/vol) Triton X-100 depending on the experiment; the total volume was 20 ml. The whole-cell bioconversion was processed at different temperatures (18, 25, 30, and 37°C) for 24 h. All experiments were carried out in triplicate.

### Analytical Methods

Cell density was measured at 600 nm at appropriate dilutions (Bausch & Lomb Spectronic 1001). The culture was diluted to the linear range with 0.95% (W/V) NaCl.

For extraction of FA and HFA during bioconversion, 1 ml of supernatant of the cell broth was recovered by centrifuging (13,780 g, 5 min). Then, 1.5 ml of chloroform and 1.5 ml of methanol containing 15% (vol/vol) sulfuric acid were added with 500 μL of 1 g/L undecylic acid as internal standards. The mixtures were mixed by vortexing. Then, the mixtures were exposed at 100°C for 4 h for derivatization. After the heating step, the mixtures were vortexed vigorously for 20 s, stood for 3 min, and operated three times. After centrifugation for 5 min at 1,666 g, the mixture in the tube separated into two layers. The organic layer was recovered by passing through a pipette containing 1 g of anhydrous sodium sulfate for GC analysis.

The concentrations of FFA and HFA in each sample were quantified by the GC-FID system (GC2014, Shimadzu Co., Japan) with a flame ionization detector (FID) and a 30-m DB-5 column (30 m × 0.25 mm × 1 m, Agilent Co., Palo Alto, CA). Nitrogen was used as the carrier gas, and the flow rate was set at 1 ml/min. The injector and detector temperatures were both set at 280°C. The oven temperature was initially held at 150°C for 1 min. Thereafter, the temperature was raised with a gradient of 10°C/min until the temperature reached 200°C, then raised with a gradient of 20°C/min until the temperature reached 240°C. This temperature was held for 3 min.

## Results and Discussion

### Biosynthesis of ω-Hydroxydecanoic Acid From Decanoic Acid Using the AlkBGT System

**Figure 1:**
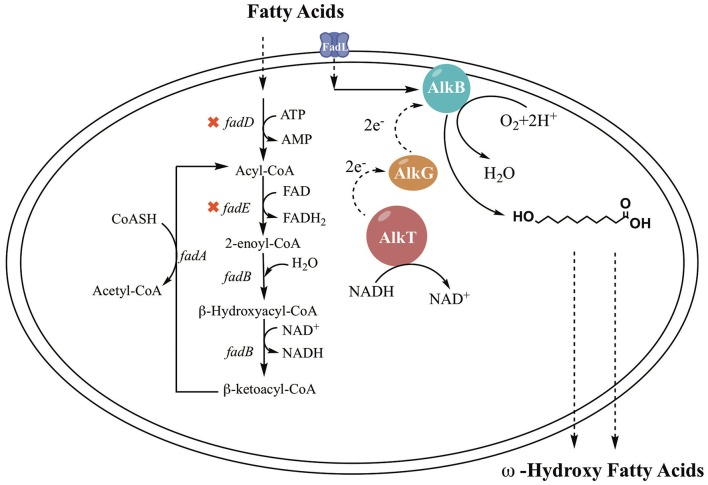
The simplified bioconversion pathways in the engineered *E. coli* strain.

In previous studies, it was demonstrated that the AlkBGT system took three steps to achieve ω-oxyfunctionalization of alkane (Jan et al., 2002). The efficiency of AlkBGT co-expression may affect the multistep reactions during ω-HFA biosynthesis. In general, ribosomal interactions with mRNA control translation initiation and the translation rate of proteins (Borujeni et al., 2014). Hence, in this experiment, the AlkBGT encoded by the *alkB, alkG*, and *alkT* genes from *P. putida* GPo1 was cloned with optimized RBS into vector pTrc99a (named pBGT) to generate the ω-HFA biosynthetic pathway (Figure 1).

**Figure 2:**
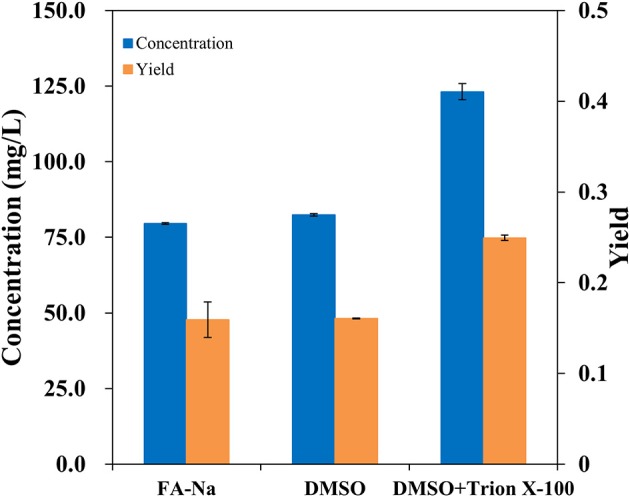
The effects of decanoic acid with differential status in the whole-cell bioconversion of W3110(pBGT) on the concentration (blue) and yield (orange) of ω-hydroxydecanoic acid production.

Traditionally, the major method for performing bioconversion is to use isolated enzymes *in vitro*; however, this method usually ignores the structural characteristics and metabolic regulation of these enzymes during the overexpression process inside the host cells. *In vivo*, most oxygenases are membrane-bound proteins and have a multi-component structure (Schrewe et al., 2013). Since AlkB is membrane-bound, it seemed to be more efficient to employ whole-cell bioconversion rather than one-pot biocatalysis *in vivo*. To verify the hydroxylation of decanoic acid in the AlkBGT system, the whole-cell bioconversion of W3110(pBGT) was investigated first using W3110(pTrc99a) as a control. In this case, considering the solubility and dispersion status of decanoic acid in the medium, we chose DMSO as the co-solvent and TritonX-100 as the surfactant. Three conditions, specifically sodium fatty acids, fatty acids dissolved in dimethyl sulfoxide (DMSO) and fatty acids dissolved in DMSO containing 10% (vol/vol) Triton X-100, were tested. Approximately 0.5 g/L decanoic acid was added in the bioconversion medium. The experiment was performed at 30°C, and the IPTG concentration was 0.1 mM. The conversion rate and concentrations of ω-hydroxydecanoic acid from W3110(pBGT) in different conditions are shown in Figure 2. No ω-hydroxydecanoic acid accumulated in W3110(pTrc99a) (data not shown); however, the strain W3110(pBGT) expressing AlkBGT had the ability to convert decanoic acid to ω-hydroxydecanoic acid. Among these three different substrates, decanoic acid dissolved in DMSO containing 10% (vol/vol) Triton X-100 showed the best performance for ω-hydroxydecanoic acid production. The concentration and yield of ω-hydroxydecanoic acid achieved 123.15 mg/L and 0.24 mol/mol, respectively. The yield was approximately 56.6% higher than that obtained using sodium decanoic acid as the substrate (Figure 2). Hence, decanoic acid dissolved in DMSO containing 10% (vol/vol) Triton X-100 was chosen in further studies. Since AlkBGT are the key enzymes of ω-hydroxydecanoic acid production in whole-cell bioconversion, the optimized expression of AlkBGT will be critical for further improvement of ω-hydroxydecanoic acid production.

### Optimization of AlkBGT Expression for ω-Hydroxydecanoic Acid Production

**Figure 3:**
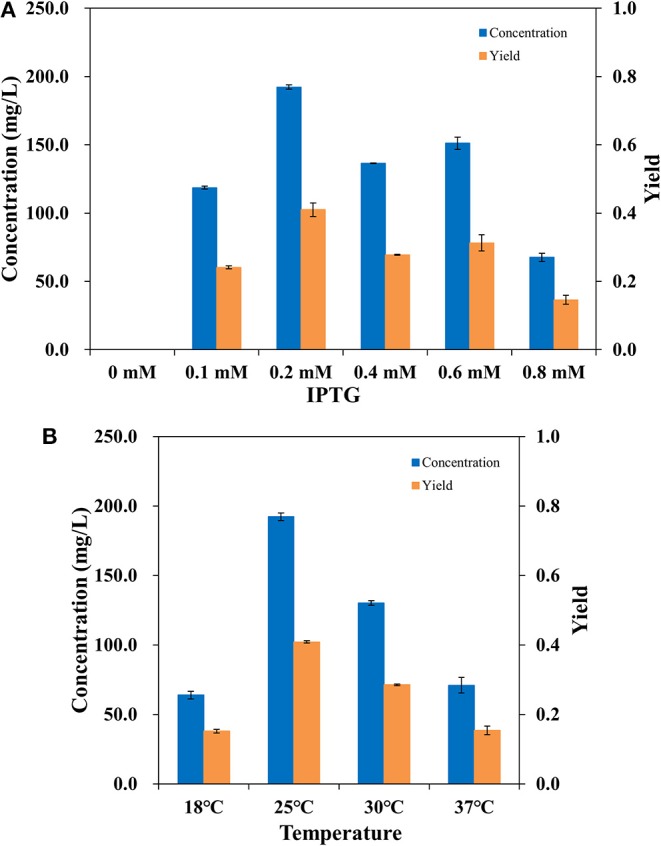
The concentration (blue) and yield (orange) of ω-hydroxydecanoic acid produced by whole-cell conversion of W3110(pBGT) cultured with different concentrations of IPTG **(A)**. The concentration (blue) and yield (orange) of ω-hydroxydecanoic acid produced at different temperatures by the whole-cell conversion of W3110(pBGT) cultured with 0.2 mM IPTG **(B)**.

To make the whole-cell system more efficient, the expression level of AlkBGT in W3110(pBGT) was optimized. Different concentrations of IPTG, 0.1, 0.2, 0.4, 0.6, and 0.8 mM, were tested in the cultivation conditions. The culture without the addition of IPTG was selected as the control. Whole-cell bioconversion was performed at 25°C. The yields and concentrations of ω-hydroxydecanoic acid in W3110(pBGT) collected following cultivation with different IPTG concentrations are shown in Figure 3A. With 0 mM IPTG addition, no ω-hydroxydecanoic acid accumulated due to the low expression of AlkBGT. The ω-hydroxydecanoic acid yields of whole cells induced by 0.1, 0.4, and 0.6 mM IPTG reached 0.24, 0.27, and 0.31 mol/mol, respectively. The highest concentration of ω-hydroxydecanoic acid was produced with the addition of 0.2 mM IPTG and reached 192.22 mg/L with the highest yield of 0.41 mol/mol (Figure 3A). When the concentration of IPTG increased to 0.8 mM, the yield of ω-hydroxydecanoic acid dropped dramatically to only approximately 0.15 mol/mol, which was only 36.6% of that of 0.2 mM IPTG. Hence, this result indicated that 0.2 mM IPTG was the optimum inducing concentration for AlkBGT overexpression in W3110(pBGT), and it was applied in further whole-cell bioconversion experiments.

Incubation temperature affects the catalysis of enzymes during the process of whole-cell bioconversion. To further improve the biocatalysis of AlkBGT in whole-cell bioconversion, four different temperatures, 18, 25, 30, and 37°C, were investigated during bioconversion. The yields and concentrations of ω-hydroxydecanoic acid of W3110(pBGT) in different temperatures are shown in Figure 3B. When whole-cell bioconversion was performed at 18 and 37°C, the concentrations of ω-hydroxydecanoic acid were similar, approximately 63.92 and 70.98 mg/L, respectively, with low yields of ω-hydroxydecanoic acid. At 30°C, the concentration and yield of ω-hydroxydecanoic acid on decanoic acid increased to 130.1 mg/L and 0.28 mol/mol, which were approximately two times higher than those at 18°C. The best result was achieved at 25°C among all of these different temperatures. The highest concentration and yield of ω-hydroxydecanoic acid on decanoic acid reached 192.22 mg/L and 0.41 mol/mol (Figure 3B). Therefore, 25°C was the optimum temperature for whole-cell bioconversion and was applied for further studies.

### Further Genetic Modification of the FA Degradation Pathway

**Figure 4:**
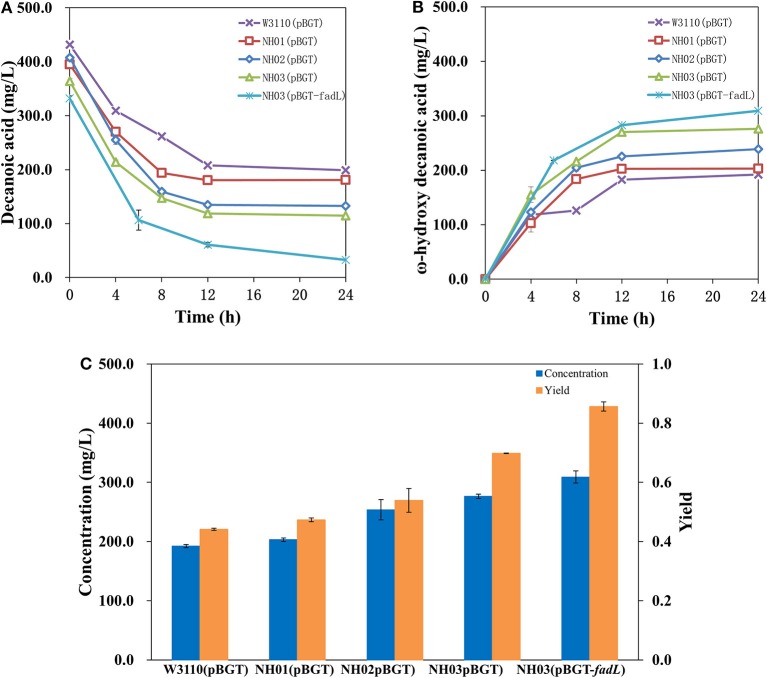
Profiles of decanoic acid (C10FA) consumption and ω-hydroxydecanoic acid (C10HFA) accumulation in the whole-cell bioconversion of W3110(pBGT), NH01(pBGT), NH02(pBGT), NH03(pBGT), and NH03(pBGT-*fadL*). **(A)** The concentration of decanoic acid, **(B)** the concentration of ω-hydroxydecanoic acid. The summary of the concentration (blue) and yield (orange) of ω-hydroxydecanoic acid at the final time point in different engineered strains **(C)**.

In a previous study, it was demonstrated that single deletion of *fadD* and *fadE* or double deletion showed high enhancement of FA accumulation (Steen et al., 2010; Li et al., 2012; Cao et al., 2016; Jawed et al., 2016). Hence, it seemed that deletion of *fadE* and *fadD* in *E. coli* might block the β-oxidation of decanoic acid, the precursor of ω-hydroxydecanoic acid, and further enhance the yield of ω-hydroxydecanoic acid. Engineered strains NH01 (Δ*fadE*), NH02 (Δ*fadD*), and NH03 (Δ*fadE* Δ*fadD*) were constructed via a CRISPR-Cas9-based approach. pBGT was transformed into NH01, NH02 and NH03 and formed NH01(pBGT), NH02(pBGT), and NH03(pBGT), respectively. The yields and concentrations of ω-hydroxydecanoic acid of these strains are shown in Figure 4. As a result, higher yields of ω-hydroxydecanoic acid were found in these three mutants than in W3110(pBGT). The yield of ω-hydroxydecanoic acid on decanoic acid in NH01(pBGT) achieved 0.47 mol/mol, which was 14.6% higher than that in W3110(pBGT); however, it was 13% lower than that in NH02(pBGT) (0.54 mol/mol). The deletion of *fadD* seemed to prevent decanoic acid degradation more efficiently than the deletion of *fadE*. This result was also consistent with a previous study (Bae et al., 2014). This result might be explained because NH01 with only *fadE* knockout was still able to synthesize Acyl-CoA from FA, and then Acyl-CoA might be utilized further by other metabolic pathways. The highest yield of ω-hydroxydecanoic acid was found in NH03(pBGT) with deletions of both *fadE* and *fadD*, reaching 0.70 mol/mol, which was 70.7% higher than that of W3110(pBGT). Deleting the key genes *fadE* and *fadD* related to the β-oxidation cycle showed large effects on ω-hydroxydecanoic acid accumulation during whole-cell bioconversion due to the reduction of the catabolic degradation of decanoic acid.

### Effect of Native *Fadl* Overexpression on Enhanced Decanoic Acid Consumption and ω-Hydroxydecanoic Acid Accumulation

Uptake of substrates, especially hydrophobic molecules, into cells through transporter proteins is critical and is one of the limiting steps in whole-cell bioconversion. It was proven by isotopic labeling that the deletion of *fadD* decreased FA transport levels (Weimar et al., 2002). Therefore, an important aspect of enhancing catalysis is to improve transport ability. In this study, overexpression of the transporter AlkL also showed no effect on ether decanoic acid consumption or ω-hydroxydecanoic acid accumulation (data not shown). The outer membrane protein FadL of *E. coli* plays an important role in importing FAs into the cell, and FadL allows the entry of small hydrophobic molecules into the outer membrane through a hydrophobic channel (Call et al., 2016). It was reported that overexpression of *fadL* resulted in increased conversion of long-chain fatty acids to ω-hydroxy long-chain fatty acids (Bae et al., 2014). To determine whether FadL might increase catalysis for ω-hydroxydecanoic acid production, the plasmid pBGT-*fadL* was constructed. The profiles of decanoic acid consumption and ω-hydroxydecanoic acid accumulation in whole-cell bioconversion of NH03(pBGT-*fadL*) are shown in Figure 4C. After 24 h of whole-cell bioconversion, 308.98 mg/L ω-hydroxydecanoic acid was observed, and the yield of ω-hydroxydecanoic acid on decanoic acid reached 0.86 mol/mol. The yield of ω-hydroxydecanoic acid on decanoic acid in NH03(pBGT-*fadL*) increased by ~22.8 and 110% relative to yields of the strains NH03(pBGT) and W3110(pBGT), respectively (Figure 4C). FadL overexpression increased the concentration and yield of ω-hydroxydecanoic acid, which might be because the expression of the transporter FadL enhanced the availability of decanoic acid for the AlkBGT hydroxylation system inside the cell. It was reported that a *Pseudomonas aeruginosa* outer membrane protein, ExFadLO, which belongs to the family of FadL proteins, was involved in the export of long-chain oxygenated fatty acids. According to a 3D model structure, ExFadLO had similar features and structural conformation to those described for previously crystallized FadL transporters from *E. coli* (Martínez et al., 2013). However, no conclusive evidence has been obtained for this hypothesis.

### Biosynthesis of ω-Hydroxyoctanoic Acid and ω-Hydroxydodecanoic Acid

**Figure 5:**
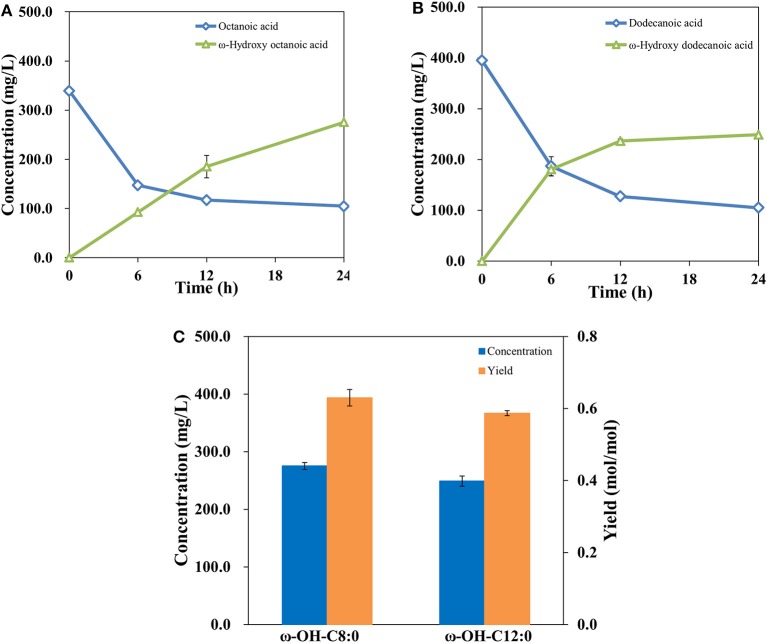
Profiles of octanoic acid (C8FA) consumption and ω-hydroxy octanoic acid (C8HFA) accumulation in whole-cell bioconversion of NH03(pBGT-*fadL*) **(A)**. Profiles of dodecanoic acid (C12FA) consumption and ω-hydroxy dodecanoic acid (C12HFA) accumulation in whole-cell bioconversion of NH03(pBGT-*fadL*) **(B)**. Summary of the concentrations (blue) and yields (orange) of ω-hydroxyoctanoic acid and ω-hydroxydodecanoic acid at the final time point in NH03(pBGT-*fadL*) **(C)**.

The study above suggested that a ω-hydroxydecanoic acid biosynthesis system with high efficiency has been established. It was demonstrated that AlkBGT is known to catalyze the oxyfunctionalization of medium-chain alkanes and fatty acids (Kusunose et al., 1964; Eggink et al., 1987). To further explore the substrate spectrum of this engineered whole-cell catalysis system of NH03 (pBGT-*fadL*), octanoic acid and dodecanoic acid, which are medium-chain fatty acids, were employed as substrates. The results are shown in Figures 5A,B. The bioconversion process of octanoic acid and dodecanoic acid was similar to that of decanoic acid. In the initial phase, highly increased product concentrations and decreased substrate levels were observed, whereas after 12 h of incubation, the rates of product accumulation and substrate consumption became slow. At 24 h, the accumulation of ω-hydroxyoctanoic acid reached 275.48 mg/L, and the yield of ω-hydroxyoctanoic acid on octanoic acid was 0.63 mol/mol. The accumulation of ω-hydroxydodecanoic acid reached 249.03 mg/L with a yield of 0.59 mol/mol (Figure 5C). It was noted that there are differences in efficiency among different fatty acid chain lengths. This phenomenon is consistent with a previous report, and it may be attributed to a specific amino acid position in the substrate-binding pocket of AlkB that determines the length of substrate (van Beilen et al., 2005). In the process of substrate uptake, small hydrophobic compounds can easily diffuse into the cell, but large hydrophobic molecules might be restrained by the outer membrane to some extent. However, most hydrophobic substrates are often toxic to living cells, and toxic substances that are detrimental impact bioconversion negatively, which is the major drawback in production (Scheps et al., 2013). Thereby, the lower bioconversion efficiency of octanoic acid than decanoic acid was probably caused by the variable toxicity of different chain lengths of fatty acids that accumulated in cells.

## Conclusion

In this study, several strategies were applied to enhance the whole-cell bioconversion of strains, including optimized overexpression of *alkBGT* from *P. putida* GPo1, deletion of the FA β-oxidation-associated key enzymes FadE and FadD, and overexpression of the native FadL. The concentrations of ω-hydroxyoctanoic acid, ω-hydroxydecanoic acid and ω-hydroxydodecanoic acid in NH03(pBGT-*fadL*) reached 275.48, 308.98, and 249.03 mg/L, respectively. The yields reached 0.63, 0.86, and 0.56 mol/mol, respectively. This study demonstrated the great potential of these engineered strains for the production of ω-hydroxy medium-chain fatty acids from medium-chain fatty acids by whole-cell bioconversion.

## Data Availability Statement

All datasets generated for this study are included in the manuscript/supplementary files.

## Author Contributions

HW, GB, and K-YS designed the experiments. QH performed the research experiments. QH, GB, K-YS, and HW analysis the data. QH and HW wrote the manuscript. All authors read and approved the final manuscript.

### Conflict of Interest

The authors declare that the research was conducted in the absence of any commercial or financial relationships that could be construed as a potential conflict of interest.
